# Proportion and associated factors of respectful maternity care during childbirth in North Showa zone public health institutions, North Showa, Ethiopia: An institutional-based cross-sectional study

**DOI:** 10.3389/fpubh.2022.878019

**Published:** 2022-07-29

**Authors:** Nakachew Sewnet Amare, Abebayehu Melesew Mekuriyaw, Getaye Worku Tesema, Yeshinat Lakew Ambaw

**Affiliations:** ^1^Clinical Midwifery, Department of Midwifery, College of Medicine and Health Sciences, Debre Berhan University, Debre Berhan, Ethiopia; ^2^Maternity and Reproductive Health, Department of Midwifery, College of Medicine and Health Sciences, Debre Berhan University, Debre Berhan, Ethiopia

**Keywords:** childbirth, Ethiopia, labor and delivery, respectful maternity care, proportion

## Abstract

**Background:**

Respectful maternity care is one of the key strategies to increase access to use skilled maternity care services. However, limited studies are done about the extent of respectful maternity care during labor and delivery in Ethiopia, particularly in the study area.

**Objective:**

This study aimed to determine the proportion and identify the associated factors of respectful maternity care during childbirth among women who gave birth in North Showa zone public health institutions, North Showa zone, Ethiopia, 2020.

**Methods:**

An institutional-based cross-sectional study was conducted among women who got birth in North Showa public health institution from October 20 to November 20, 2020. A systematic random sampling technique was used to select study participants. Logistic regression with adjusted odds ratio and 95% uncertainty interval was used to declare statistically significant variables based on *p* < 0.05 in the multivariable logistic regression model.

**Result:**

The overall proportion of respectful maternity care during childbirth was 48.6 % (95% CI: 44.6–52.3%). Urban residence AOR = 2.6 (95% CI: 1.8, 3.6), being multiparous AOR = 1.6 (95% CI: 1.1, 2.3), having planned pregnancy AOR = 2.4 (95% CI: 1.3, 4.3) and giving birth in health center AOR = 1.6 (95% CI: 1.2, 2.8) were statistically significant factors with respectful maternity care during labor and delivery.

**Conclusions:**

The proportion of respectful maternity care during childbirth is low. Being from an urban community, being multiparous, having planned pregnancy, and giving birth in a health center were factors that could increase the likely hood of women getting respectful maternity care during childbirth. Based on the identified factors strategies need to be designed and implemented to enhance the level of respectful maternity care.

## Background

Respectful maternity care (RMC) is an approach centered on an individual, based on principles of ethics and respect for human rights, and promotes practices that recognize women's preferences and women's and newborns' needs (1). Non-respectful maternity care during childbirth is a violation of a universal human right that is due to every childbearing woman in every health system, is common throughout the world, and can occur at the level of interaction between the woman and provider leading to low intake of maternity service (2).

In 2013 about 289,000 women died, worldwide, due to complications in pregnancy and childbirth (3). The most important intervention in reducing maternal morbidity and mortality is using skilled assistance during pregnancy and childbirth. Improving the quality of care provided, especially elements of respectful maternity care is an important strategy to increase the use of facility-based childbirth in low-income (4, 5).

Childbirth is a special moment for parents, families, and communities but can also be a time of intense vulnerability for women to be exposed to disrespectful and abusive behavior while at the health facilities. If mothers do not receive what they perceive as compassionate and respectful maternity care during facility-based childbirth their intention to use facility-based maternity services will be reduced (6).

A woman's experience of care in childbirth is an important determinant of her future decisions related to seeking health care from health facilities. Women's negative encounters with health workers during delivery could result in regret to use maternity health care services (7). However, many women across the world experience disrespectful, abusive, or neglectful treatment during childbirth in health institutions (8). Improving respectful maternity care is a critical strategy to increase facility-based childbirth and ensure effective implementation of women's rights and women-centered approaches in maternal health services.

There are factors associated with the level of respectful maternity care identified by previous studies such as sociodemographic variables, mode of delivery, and complications during labor and delivery (9, 10). This study intended to identify significant factors that can be associated with the provision of respectful maternity care agreeing to the nature of the community in the study area.

Ethiopian federal ministry of health in its Health Sector Transformation Plan (HSTP) has planned to increase the level of deliveries attended by skilled birth attendants. To achieve this plan, the Ministry has identified caring, respectful, and compassionate health professionals as one of the four transformation agendas. Lack of respect for patients and their families is a common complaint and having RMC health professionals is a critical requirement to ensure equity and achieve high-quality health services on its HSTP.

Despite the problem, little research has been conducted in Ethiopia, particularly in the study area about the proportion of respectful maternity care and its associated factors. The current study is therefore aimed to determine the magnitude and factors associated with respectful maternity care during childbirth in north Showa zone public health institutions, North Showa, Ethiopia, 2020.

## Materials and methods

### Study design and period

An institution-based cross-sectional study was conducted from October 20 to November 20, 2020.

### Source and study population

#### Source of population

The source populations for the study were all women who gave birth at public health institutions in the North Shewa zone.

#### Study population

All women who gave birth at the selected health institutions in the North Shewa zone during the data collection period.

### Sample size determination

The sample size is calculated using Epi Info Stat Calc version 7.2.1 population survey by taking assumptions of population size >10,000, 95% confidence interval, the proportion of compassionate and respectful maternity care (35.8%) from the previous study (11), confidence limit (margin of error) 5%, and by adding 10% non-response rate. The final sample size was calculated to be.


(1) $$n=\frac{{\left(z\alpha /2\right)}^{2}(p\left(1-p\right)d^{2}={\left(1.96\right)}^{2}\left(0.358\right)\left(1-0.358\right)}{{\left(0.05\right)}^{2}}=353$$

Where: - *zα*/2 is z value for 95% confidence level

d is margin of error and

p is proportion

by adding non-response rate 10% with 2 design effect (353+ 35) 2 = our final sample size was 776.

### Sampling technique and procedure

There are 10 public hospitals and 90 health centers in the North Showa zone; from those public health institutions, we selected 5 hospitals and 45 health centers randomly. The total sample sizes were allocated proportionally to each of the selected hospitals and health centers by reviewing the number of deliveries attended during the preceding month before data collection. Finally, the determined samples were selected by a systematic random sampling technique.

### Inclusion and exclusion criteria

#### Inclusion criteria

All women who gave birth at selected health institutions in the North Shewa zone during the data collection period.

#### Exclusion criteria

Women who are unable to communicate effectively due to serious illness were supposed to be excluded from the study.

### Data collection tools procedures

Data were collected using a structured and semi-structured interviewer-administered questionnaire having two parts. The first part contained baseline data regarding the Socio-demographic ideas of respectful maternity care. The remaining part of the questionnaire composes of obstetrics-related questions. The questionnaires were developed in English by reviewing various literature and translated into the local language Amharic then retranslated back to English to check the consistency. Pretest was conducted at public health institutions in the East Gojjam zone and necessary corrections to the tool were employed accordingly. Data collection was done by 35 trained midwives and supervised by 5 BSc Midwives.

### Operational definitions

- **Respectful maternity care:-** Women were considered to have received respectful maternity care during labor and childbirth if they answered yes to all of those questions assessing RMC or verification criteria used for assessing the four categories (friendly care, abuse free care, timely care, and discrimination-free care) of RMC during labor and childbirth (10, 12, 13).

### Statistical analysis

All collected questionnaires were rechecked for completeness and coded. Then these data were entered and cleaned using Epi Info 7 software and exported to SPSS version 21 for analysis. Bivariable logistic regression was employed to identify an association, and a multivariable logistic regression model was used to identify the effect of independent variables on the outcome variable.

Variables having *P* < 0.25 in the Bivariable analysis were fitted into the multivariable logistic regression model. Ninety-five percent confidence interval of odds ratio computed and variable having *P* < 0.05 in the multivariable logistic regression analysis considered as determinant factors for respectful maternity care. Before the actual logistic regression analysis, the necessary assumption of the logistic regression model was checked by using the Hosmer-Lemeshow test of goodness of fit which has a chi-square distribution.

## Results

### Socio-demographic characteristics of the participants

A total of 776 post-partum women were interviewed in this study, and all of the participants completed the questionnaire making the response rate 100%. The mean age of participants was 27.65 ± 5.2, and it ranged from 16 to 45 years. The participants' average family income was 6476.1 and the minimum and maximum family income were 200 and 80, 000 ETB, respectively). Among respondents; 634 (81.7), 731 (94.2%), 722 (93%), and 479 (61.7%), of the participants, were orthodox, married, Amhara, and lived in urban (Table 1).

**Table 1:** Socio-demographic characteristics of women who gave birth in North Showa zone public health institutions, North Showa zone, Ethiopia, 2020 (N = 776).

**Characteristics**	**Frequency**	**Percent (%)**
Age in years
15–19	22	2.8
20–24	184	23.7
25–29	316	40.7
30–34	169	21.8
>35	85	11.0
Religion
Orthodox	634	81.7
Muslim	108	13.9
Protestant	32	4.1
Catholic	2	0.3
Marital status
Single	31	4.0
Married	731	94.2
Divorced	8	1.0
Separated	6	0.8
Educational status
Can't read and write	178	22.8
Can read and write	98	12.6
Primary school	138	17.8
Secondary school	157	20.2
Diploma and above	295	26.4
Husband educational status
Can't read and write	140	18.0
Can read and write	124	16.0
Primary school	129	16.6
Secondary school	148	19.1
Diploma and above	235	30.3
Ethnicity
Amhara	722	93.0
Oromo	48	6.2
Tigre	6	0.8
Residence
Urban	479	61.7
Rural	297	38.7
Occupation
Housewife	391	50.4
Private employer	66	8.5
Government employee	157	22.6
Merchant	73	9.4
Student	25	3.2
Daily labor	16	2.1
Farmer	30	3.9
Husband occupational status
Farmer	288	37.1
Government employee	216	27.8
Merchant	160	20.6
Private employer	52	6.7
Student	8	1.0
Daily labor	28	3.6
Other *	24	3.1

*Includes deriver (18), jobseeker (3), tailor (3).

### Obstetrics/health care service-related characteristics

over half, 508 (65.5) of the respondents were multiparous, and 678 (87.4%) and 720 (92.8%) of the participants had planned and wanted pregnancy, respectively. Among the women who had ANC follow-up (715); 425 (45.5%) of them had ANC follow-up about four and above. Among women who had ANC follow-up (715), the majority 84.6 % (605) had ANC follow-up at the health center the others 19.4% (139) and 12.4 % (890) had ANC follow up at the government hospital and private health institution, respectively. The majority, 684 (88.1) of the participant's labor was established spontaneously, and the majority 502 (64.2%) of women's duration of labor was <12 h. On the other hand, three fourth of the participants stayed for more than 12 h in the HF. Nearly half and three fourth of the health care providers were females and midwives by profession. 422 (54.4%), 733 (94.5%), 613 (79.0%), and 423 (54.5%) of the respondents were delivered at night time, the neonate was alive, had no complications during labor & delivery, and had delivered in the health center, respectively (Table 2).

**Table 2:** The Obstetric/health care service-related characteristics among women who gave birth in north Showa zone public health institutions, North Showa zone, Ethiopia, 2020 (*N* = 776).

**Characteristics**	**Frequency**	**Percent (%)**
Parity
Primi parous	268	34.5
Multiparous	508	65.5
Planed pregnancy
Yes	678	87.4
No	98	12.6
Have ANC follow up
Yes	714	92.0
No	62	8.0
Labor start
Spontaneous	684	88.1
Induced	68	8.8
C/s	24	3.1
Mode of delivery
SVD	569	73.3
Instrumental	59	7.6
C/S	148	19.1
Time in labor
<12 h	502	64.7
>12 h	274	35.3
Time stays in a health facility
<12 h	249	32.1
12–24 h	291	37.5
>24 h	236	30.4
Sex of health provider attending labor
Female	357	46
Male	419	54
The profession of health provider
Midwifery	574	74
Nurse	5	0.6
General practitioner	1	0.1
Intern	29	3.7
ISO	94	12.1
Gynecology	73	9.4
Time of delivery
Day	354	45.6
Night	422	54.4
Neonate alive
Yes	733	94.5
No	43	5.5
Any complication during L&D
Yes	163	21.0
No	613	79.0
Place of delivery
Health center	423	54.5
Hospital	353	45.5

### The proportion of respectful maternity care (RMC) during childbirth

Of all the respondents, 377 (48.6%) of the participants have received respectful maternity care during childbirth. The rest 399 (51.4%) women have not received maternity care during childbirth respectfully.

### Factors associated with respectful maternity care (RMC) during childbirth

In this study binary Logistic regression was performed to assess the association of each independent variable with the outcome variable (RMC). The variables that showed a significant level (*p* < 0.25) were added to the multivariate regression model. The model contained seven (7) independent variables; namely residence, parity, planned pregnancy, the status of ANC follows up, duration of stay in the health facility, complications during labor and delivery, and place of delivery were the associated variables in bivariate Logistic regression. The researchers identified four variables that were significantly associated with RMC.

The result of this study revealed that those respondents who were from urban were 2.6 times more likely to receive respectful maternity care (RMC) than those who were from rural, [AOR = 2.6 (95% CI: 1.8, 3.6)]. Those women who were multiparous also received respectful maternity care 1.6 times higher than those who were primiparous women, [AOD = 1.6 (95% CI: 1.1, 2.3)]. Similarly, those participants who had planned pregnancy had over two times the odds to receive RMC than those who were unplanned pregnancy, [AOR = 2.4 (95 % CI: 1.3, 4.3)].

Respondents who were delivered at the health center were 1.6 times more likely to receive respectful service than those mothers delivered at the hospital, [AOR = 1.6 (95% CI: 1.1, 2.3)].

On the other hand, the other three (3) independent variables such as status of ANC follow-up, duration of stay in the health facility, and Complications during labor& delivery were not showed significant association with our dependent variables (Table 3).

**Table 3:** Bivariate and multivariate logistic regression analysis of RMC among women who gave birth in north Showa zone public health institutions, North Showa zone, Ethiopia, 2020 (N = 776).

**Variables**	**RMC**	**COR (95% CI)**	**AOR (95%CI)**
	Yes	No		
Residence
Urban	276 (57.6)	203 (42.4)	2.6 (1.9, 3.5)	2.6 (1.8, 3.6)*
Rural	101 (34)	196 (66)	1	1
Parity
Primi parous	115 (42.9)	153 (57.1)	1	1
Multiparous	262 (51.5)	246 (48.4)	1.4 (1.05,1.9)	1.6 (1.1, 2.3)*
Planned pregnancy
Yes	352 (52)	326 (48)	3.1 (1.9, 5.0)	2.4 (1.3, 4.3)*
No	25 (25.5)	73 (74.5)	1	1
ANC follow up
Yes	367 (50.8)	352 (49.2)	1	1.2 (0.6, 2.5)
No	15 (24.1)	47 (75.9)	3.2 (1.7, 5.8)	1
Stay in a health facility
<12 h	115 (46.2)	134 (53.8)	1.2 (0.8, 1.7)	1.0 (0.7, 1.6)
12–24 h	166 (57)	125 (42)	1.9 (1.3, 2.7)	1.5 (1.0, 2.2)
>24 h	96 (40.7)	140 (59.3)	1	1
No				
Complications during labor & delivery
Yes	68 (41.7)	95 (58.2)	1	1
No	309 (50.4)	304 (49.5)	1.4 (1.01, 2.01)	1.1 (0.7, 1.7)
Place of delivery
Health center	240 (56.7)	183 (43.7)	2.0 (1.5, 2.7)	1.6 (1.1, 2.3)*
Hospital	137 (38.8)	225 (61.2)	1	1

Note that ^*^ are variables showed significant association with p ≤ 0.05.

## Discussion

This study assessed the proportion and associated factors of respectful maternity care during labor and delivery among women who gave birth in north Showa zone public health institutions, North Showa zone, Ethiopia. And the result showed that the proportion of respectful maternity care was 48.6%. This result was lower compared to the study conducted in Bahir Dar (57%) (10) and in referral hospitals of Northwest Amhara (56.3%) (9). The possible reason for this difference might be because those covered a limited area that had a limited number of health institutions.

On the other hand, this finding was higher compared to the results of the study done in Addis Ababa (21.4%) (12), Oromia (35.8%), India (28.7%) (14), Iran (24.3%) (15). The reason for this discrepancy might be some of those studies were focused on a single city and most of them included hospitals nearly equal to or over the number of selected health centers that had relatively more case flows that resulted to have more disrespectful care.

Regarding factors associated with respectful maternity care during childbirth, women who reside in urban were 2.6 times more likely to receive maternity care during labor and delivery respectfully than women who come from rural. These findings were supported by the study conducted in Bahir Dar (10). This could be explained by, even though rural women are vital, they had relatively compromised awareness, involvement, and capabilities for decision making which make them passive and non-empowered for the care they received.

Being multiparous had a positive association with respectful maternity care. Multiparous women were 1.6 times more likely to have respectful maternity care than primiparous. This result is in line with the study done in India (14). This might be due to the reason that multiparous women had the experience of giving birth and they knew what would have been expected from them and health care providers to have harmonious interaction for receiving the care respectfully.

This study also revealed that women who had planned pregnancy had over two times more chances to receive respectful maternity care. A similar finding was reported from studies done in the Oromia region (11). This might be because having a planned or timed pregnancy could inspire the women to use other maternity health care services starting from antenatal care, this might increase women's positive cooperation with the health care provider to have respectful maternity care during childbirth.

The result of the current study showed that the odds of having respectful maternity care were 1.6 times higher among women who gave birth in a health center than in a hospital. There are studies explored in line with this association, studies done in Oromia (11). The possible explanation might be due to health centers having relatively lower case flow than hospitals, this may be good for health care providers not to be bored and disrespect the laboring women.

Overall, the practice of respectful maternity care during labor and delivery in public health institutions in the North Showa Zone was low; on the other hand, the proportion of disrespectful and abusive care among study participants was 51.4% which is higher compared to previous findings. Strategies must be designed to provide respectful maternity care for all laboring women, such as providing training for health care workers about the importance of RMC, creating a comfortable and attractive working environment for a health worker to build a harmonious interaction with women, and employing sufficient professionals on health institutions are the least of interventions to increase the proportion of the women who received respectful maternity care (RMC) during labor and delivery.

## Conclusions

The results of this study show that the proportion of respectful maternity care during labor and delivery in public health institutions in the North Showa Zone was 48.6%. On the other hand, 51.4% of the study participants faced disrespectful and abusive care.

Factors such as; urban residence, being multiparous, having planned pregnancy, and giving birth in a health center were positively associated with respectful maternity care during childbirth. Based on factors associated with RMC, specific strategies and interventions should be designed to increase the magnitude of respectful maternity care during childbirth.

### Strength and limitation of the study

- A causal relationship cannot be established due to the cross-sectional nature of the study.
- Representative sample token among women giving birth in North Showa Zone public health institutions.

## Data availability statement

The raw data supporting the conclusions of this article will be made available by the authors, without undue reservation.

## Ethics statement

The studies involving human participants were reviewed and approved by Debre Berhan University, College of Health Science Research Committee. The patients/participants provided their written informed consent to participate in this study.

## Author contributions

NA and AM designed the study, participated in the data collection, performed analysis and interpretation of data, and drafted the paper and the manuscript. GT and YA designed, approved the proposal with some revisions, participated in data analysis, and revised subsequent drafts of the paper and the manuscript. All authors contributed to the article and approved the submitted version.

## Conflict of interest

The authors declare that the research was conducted in the absence of any commercial or financial relationships that could be construed as a potential conflict of interest.

## Publisher's note

All claims expressed in this article are solely those of the authors and do not necessarily represent those of their affiliated organizations, or those of the publisher, the editors and the reviewers. Any product that may be evaluated in this article, or claim that may be made by its manufacturer, is not guaranteed or endorsed by the publisher.
